# ASPP1 deficiency promotes epithelial-mesenchymal transition, invasion and metastasis in colorectal cancer

**DOI:** 10.1038/s41419-020-2415-2

**Published:** 2020-04-08

**Authors:** Dian Liu, Ayse Ertay, Charlotte Hill, Yilu Zhou, Juanjuan Li, Yanmei Zou, Hong Qiu, Xianglin Yuan, Rob M. Ewing, Xin Lu, Hua Xiong, Yihua Wang

**Affiliations:** 1 Department of Oncology, Tongji Hospital, Tongji Medical College, Huazhong University of Science and Technology, 430030 Wuhan, China; 2 Biological Sciences, Faculty of Environmental and Life Sciences, University of Southampton, Southampton, SO17 1BJ UK; 3 Institute for Life Sciences, University of Southampton, Southampton, SO17 1BJ UK; 4 Ludwig Institute for Cancer Research, Nuffield Department of Clinical Medicine, University of Oxford, Oxford, OX3 7DQ UK; 5 NIHR Southampton Biomedical Research Centre, University Hospital Southampton, Southampton, SO16 6YD UK

**Keywords:** Cell migration, Cancer

## Abstract

The apoptosis-stimulating protein of p53 (ASPP) family of proteins can regulate apoptosis by interacting with the p53 family and have been identified to play an important role in cancer progression. Previously, we have demonstrated that ASPP2 downregulation can promote invasion and migration by controlling β-catenin-dependent regulation of ZEB1, however, the role of ASPP1 in colorectal cancer (CRC) remains unclear. We analyzed data from The Cancer Genome Atlas (TCGA) and coupled this to in vitro experiments in CRC cell lines as well as to experimental pulmonary metastasis in vivo. Tissue microarrays of CRC patients with information of clinical-pathological parameters were also used to investigate the expression and function of ASPP1 in CRC. Here, we report that loss of ASPP1 is capable of enhancing migration and invasion in CRC, both in vivo and in vitro. We demonstrate that depletion of ASPP1 could activate expression of Snail2 via the NF-κB pathway and in turn, induce EMT; and this process is further exacerbated in *RAS*-mutated CRC. ASPP1 could be a prognostic factor in CRC, and the use of NF-κB inhibitors may provide new strategies for therapy against metastasis in ASPP1-depleted CRC patients.

## Introduction

The Apoptosis Stimulating Proteins of p53 (ASPP) family consists of three members (ASPP1, ASPP2 and iASPP), which have similar sequences in the C-termini including Ankyrin repeats, SH3 domain and Proline-rich region^1–4^. Functionally they bind with apoptosis regulating proteins, including p53, p63 and p73, to regulate cell apoptosis^4^. Studies have also linked the ASPP family to a number of other processes including autophagy, RAS-induced senescence, gestational trophoblastic disease and the development of cancer^3,5–11^.

A number of studies have identified a link between members of the ASPP family, epithelial-mesenchymal transition (EMT) and cancer progression. EMT is a cell plasticity program where epithelial cells lose their cell polarity and cell–cell adhesion to gain migratory and invasive properties in turn becoming mesenchymal cells^12,13^. It is widely regarded as being crucial for cancer progression^14^. EMT-Transcription Factors (EMT-TFs) including Snail1/2 (*SNAI1*/*2)*, Twist-related protein 1/2 (*TWIST1*/*2*) and zinc-finger E-box-binding homeobox 1/2 (*ZEB1*/*2*) are able to activate the EMT program^15,16^.

The ASPP family have been implicated in cancer, ASPP1/2 are tumour suppressors, while iASPP is an oncogene^4^. High ASPP2/low iASPP expression in a number of human cancers has been evaluated and found to correlate with higher survival rates, improved curative effect and better prognosis^17–22^. Meanwhile, ASPP1 has been found to be downregulated in a variety of human cancers including acute lymphoblastic leukemia, breast cancer, hepatitis B virus-positive hepatocellular carcinoma, clear cell renal cell carcinoma and colorectal cancer (CRC)^21–25^. Previously, we have demonstrated that ASPP2 downregulation can promote invasion and migration by controlling β-catenin-dependent regulation of ZEB1^26^; however, the mechanisms underlying ASPP1’s functions in CRC remain unclear. Here, we show that reduced ASPP1 expression in CRC tissue correlates with more invasive disease. Further, we demonstrate that downregulation of ASPP1, can facilitate invasion and migration, via the NF-κB pathway, in turn activating Snail2, inducing EMT. This process is further exaggerated in *RAS*-mutated CRC. This study could provide potential prognostic indicators and novel treatments for the treatment against metastasis in ASPP1-depleted CRC patients.

## Results

### Expression of ASPP1 is decreased in colorectal tissues, and correlates with both clinical and lymph node stages

To determine the role of ASPP1 in CRC, we first investigated the protein expression of ASPP1 in 86 paired CRC and adjacent normal tissues by IHC. We demonstrated that ASPP1 is expressed in the nucleus and cytoplasm, both in normal and CRC tissue. However, we determined that ASPP1 expression was significantly lower in both the nucleus (*P* = 0.0007) and cytoplasm (*P* < 0.0001) in CRC, compared to adjacent normal tissue (Supplementary Fig. S1).

**Fig. 1:**
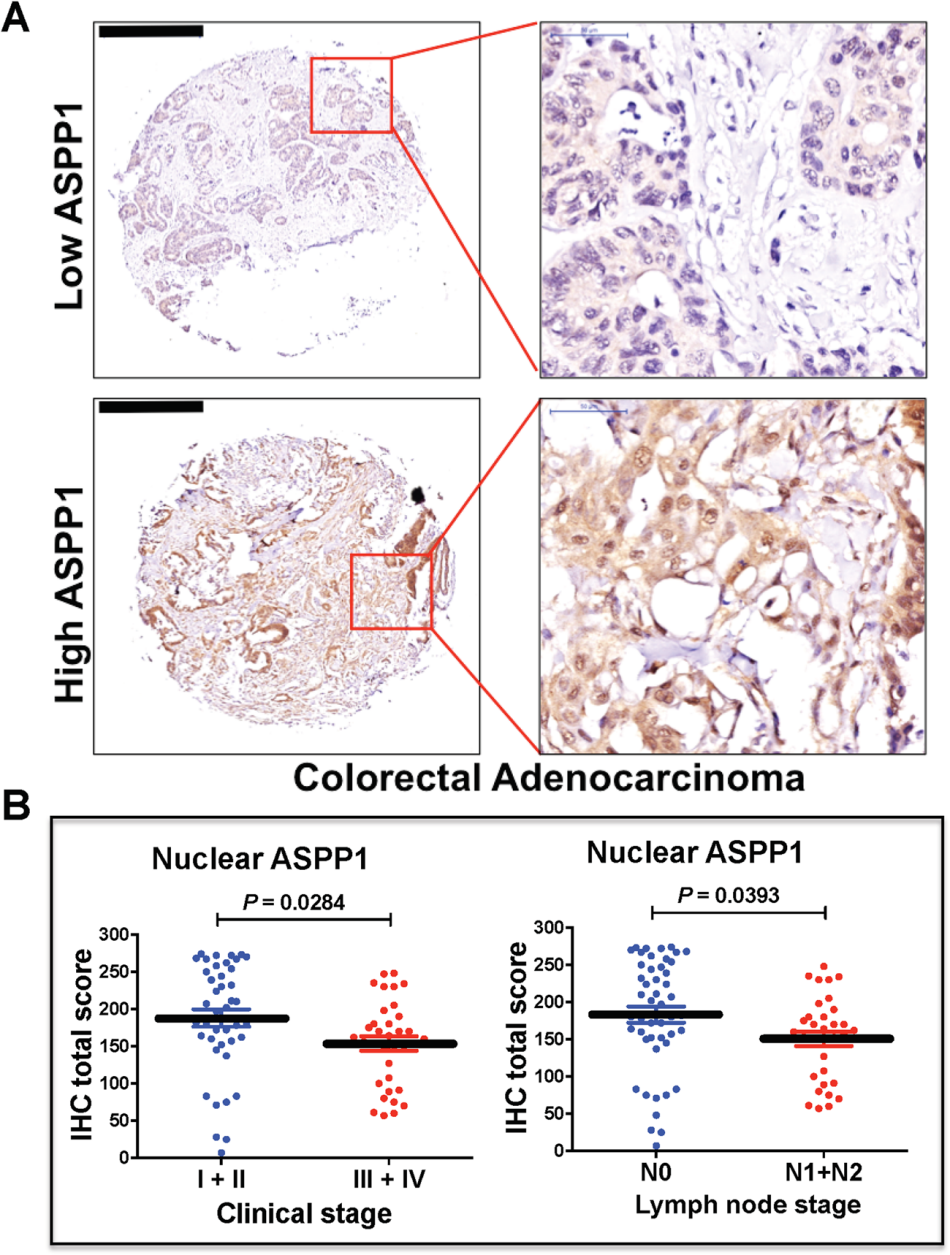
ASPP1 expression levels associate with clinical and lymph node stages in colorectal cancer (CRC). **a** Representative ASPP1 staining pattern (high or low ASPP1) in 86 tissue microarray cores. Scale bars: 500 μm. **b** The relationship between nuclear ASPP1 expression and clinical or lymph node stages in CRC samples was analysed, with *P-*values of 0.0284 and 0.0393, respectively.

Next, we analyzed the correlation between ASPP1 expressions (Fig. 1a) and clinical–pathological factors by categorizing tissues into high and low expression groups according to both clinical stage and lymph node stage. Low nuclear, but not cytoplasmic, expression of ASPP1 was significantly correlated with both lymph node metastasis and higher clinical stage (Fig. 1b; Tables 1 and 2), indicating that lower nuclear ASPP1 expression may promote invasion and migration in CRC.

**Table 1:** The relationship between patients’ clinical–pathological characteristics and nuclear ASPP1 expression in CRC.

Parameters	No.	Nuclear low	Nuclear high	*P* value
Age (years)				
≤60	32	17	15	0.54
>60	54	25	29	
Gender				
Male	48	21	27	0.127
Female	38	22	16	
Lymph node				
N0	48	18	30	0.021^a^
N1 + N2	33	21	12	
Clinical stage				
I + II	42	14	28	0.01^a^
III + IV	36	23	13	
Pathological staging				
I + II	61	29	32	0.476
III	25	14	11	
Distant metastasis				
M0	83	40	43	0.529
M1	3	2	1	
Ki67				
0	10	3	7	0.178
0.5–3	76	40	36	

*P*-values were generated by Chi-square test or Fisher’s exact test.

^a^*P* < 0.05 was considered as statistically significant.

**Table 2:** The relationship between patients’ clinical–pathological characteristics and cytoplasmic ASPP1 expression in CRC.

Parameters	No.	Cytosol low	Cytosol high	*P* value
Age (years)				
≤60	32	15	17	0.54
>60	54	29	25	
Gender				
Male	48	21	27	0.193
Female	38	22	16	
Lymph node				
N0	48	22	26	0.299
N1 + N2	33	19	14	
Clinical stage				
I + II	42	18	24	0.108
III + IV	36	22	14	
Pathological staging				
I + II	61	31	30	0.812
III	25	12	13	
Distant metastasis				
M0	83	42	41	0.584
M1	3	2	1	

*P*-values were generated by Chi-square test or Fisher’s exact test.

*P* < 0.05 was considered as statistically significant.

### ASPP1 inhibition promotes CRC invasion and migration in vivo and in vitro

**Fig. 2:**
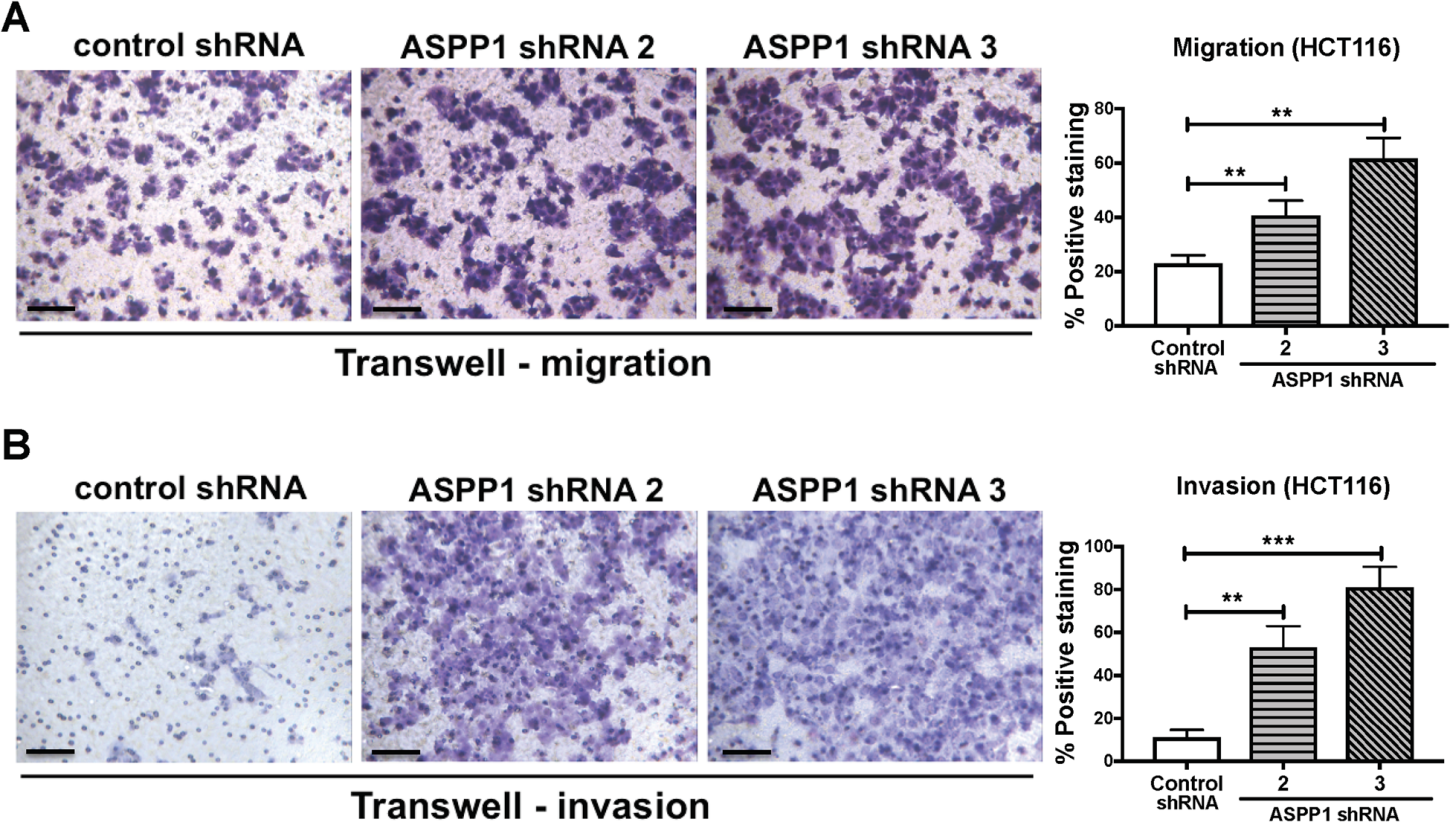
Downregulation of ASPP1 promotes cell migration and invasion in vitro. **a** Transwell migration assays in HCT116 cells with indicated treatment. Cells were stained by crystal violet. Data are mean ± s.d. *n* = 3. **P < 0.01. **b** Transwell invasion assays in HCT116 cells with indicated treatment. Cells were stained by crystal violet. Scale bar: 100 μm. Data are mean ± s.d. *n* = 3. **P < 0.01. ***P < 0.001.

Given the clinical findings implicating ASPP1, together previous studies demonstrating a role for the ASPP family in migration and invasion^26^; we sought to investigate whether ASPP1 is capable of facilitating invasion and migration in CRC. We stably expressed control or ASPP1 (*PPP1R13B*) shRNA (shRNA1, shRNA2, shRNA3, Supplementary Fig. S2) in HCT116 cells and then utilized Transwell invasion and migration assays. In both Transwell migration and invasion assays, depletion of ASPP1 significantly promoted invasion and migration in HCT116 cells (Fig. 2). Similar results have been demonstrated in a MCF10A derivative cell line, MCF10A ER:HRASV12, which was engineered to express ER:HRAS. Addition of 4-hydroxytamoxifen (4-OHT) acutely activates the RAS pathway in this cell line. MCF10A is an immortalized human breast epithelial cell line, it maintains many features of normal breast epithelial cells, including the ability to form cell–cell junctions^26^. These results show that when ASPP1 RNAi-transfected 4-OHT-treated MCF10A ER:HRASV12 cells are cultured in Matrigel, they have longer protrusions compared to those treated with 4-OHT alone (Supplementary Fig. S3), suggesting that ASPP1 inhibits RAS-induced invasion.

**Fig. 3:**
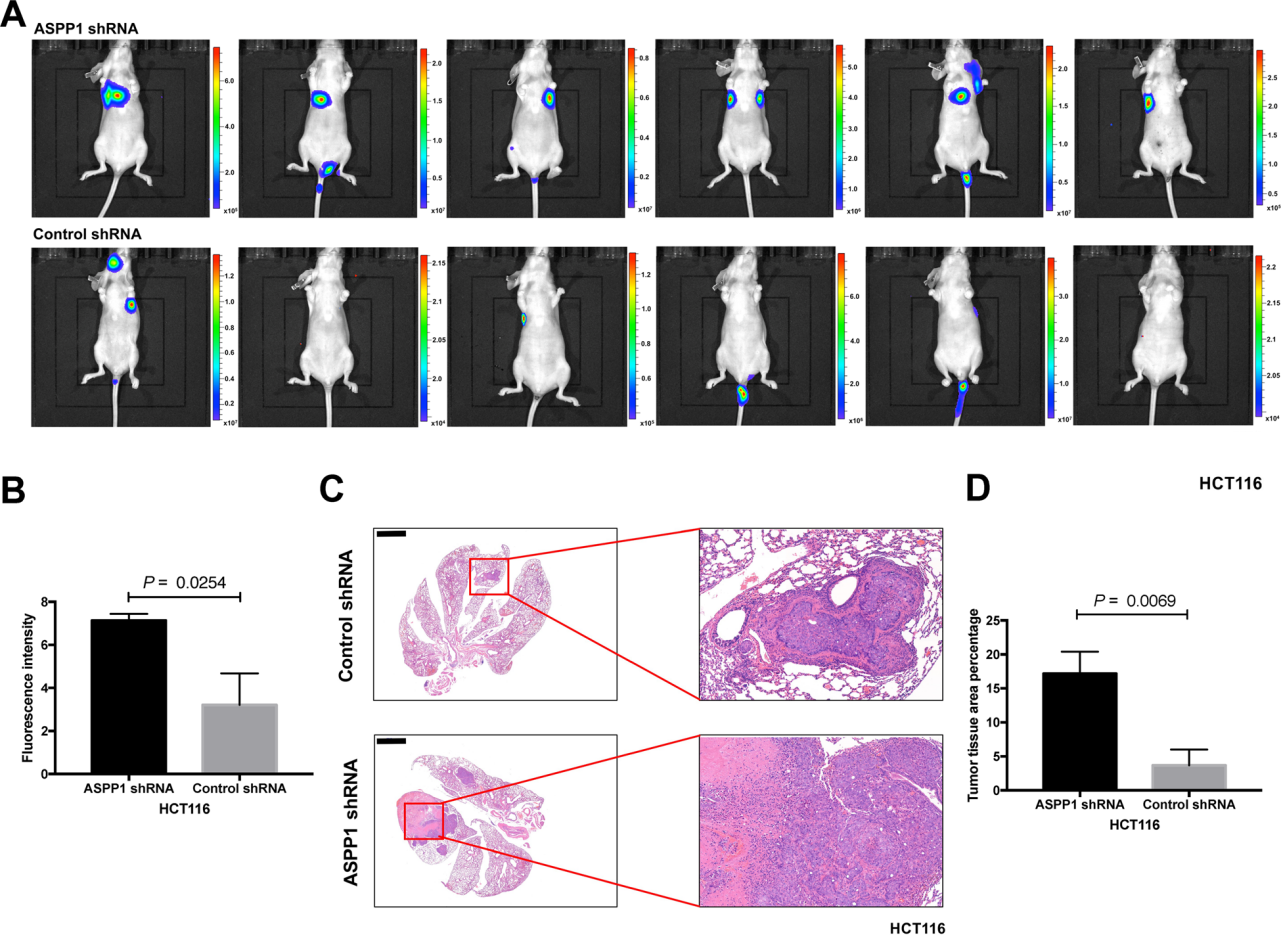
Downregulation of ASPP1 promotes lung metastasis in vivo. Bioluminescent imaging (**a**) and quantification of photon flux (**b**) of lung metastatic tissues from nude mice with intravenous injection of the indicated cells (*P* = 0.0254). The color bars in (**a**) represent bioluminescent signal in radiance (p/sec/cm2/sr). **c** H&E staining of the dissected lungs from the nude mice in the indicated group are presented (*n* = 6 mice per group), showing that ASPP1 shRNA/HCT116 cells gave rise to more and larger metastases than did control ones (*P* = 0.0069) (**d**). Scale bar: 2 mm.

Next, we conducted experiments in a mouse model to examine whether ASPP1-depleted cells have more metastatic potential by using tail vein injection of firefly luciferase tagged HCT116 cells with ASPP1 shRNA or control shRNA. Lung fluorescence signals of mice from ASPP1 shRNA group were significantly more intense, suggesting more metastasis in mice lungs (*P* = 0.0254; Fig. 3a, b). The area of metastatic clusters present in the lungs of mice injected with ASPP1 shRNA/HCT116 cells was significantly higher than the group injected with control shRNA/HCT116 cells, as examined by hematoxylin and eosin (H/E) staining (*P* = 0.0069; Fig. 3c, d). These results indicate that ASPP1 may play a critical role in inhibiting invasion and metastasis of colorectal cancer cells.

### **ASPP1 depletion induces Snail2 expression and facilitates oncogenic RAS-induced** EMT

**Fig. 4:**
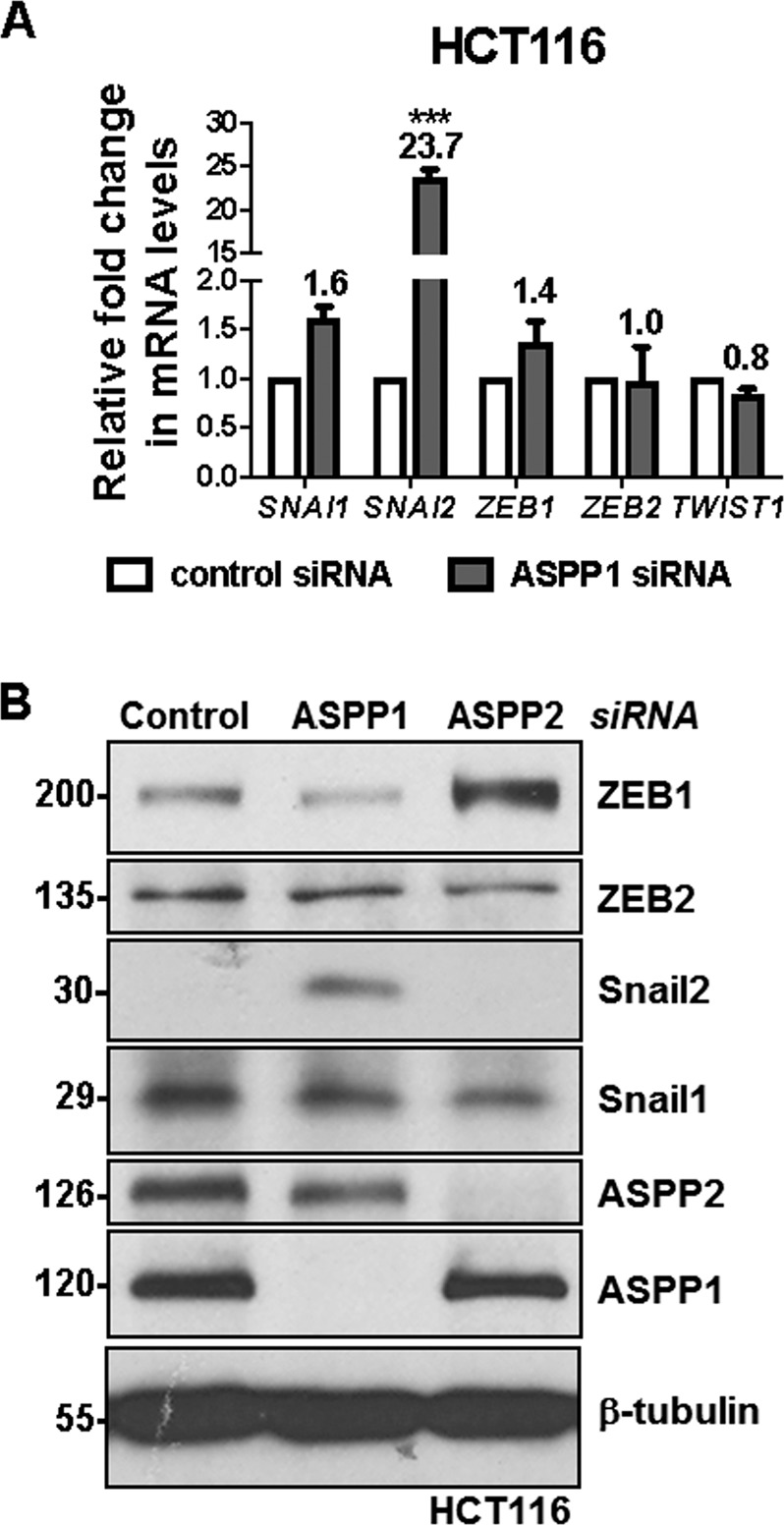
ASPP1 depletion induces Snail2 expression. **a** Relative fold change in mRNA levels of *SNAI1* (Snail1), *SNAI2* (Snail2), *ZEB1*, *ZEB2*, *TWIST1* in HCT116 cells transfected with ASPP1 siRNA and control siRNA. ***P < 0.001. Scores above the bars are relative levels when compared with control cells. **b** Protein expression of ZEB1, ZEB2, Snail2, Snail1, ASPP2 and ASPP1 in HCT116 cells with indicated treatment. β-tubulin was used as a loading control.

Previously, we identified ASPP2 as a molecular switch of epithelial plasticity via the β-catenin-ZEB1 pathway^26^. Given that the structure and function of ASPP1 is similar to ASPP2, we hypothesized they may act in a similar manner. As such, we analyzed the expression of a number of EMT-TFs in HCT116 cells transfected by siRNA against ASPP1 or control siRNA. Surprisingly, levels of Snail2 (*SNAI2*), protein and mRNA, but not those of ZEB1 or other EMT-TFs, were significantly higher upon ASPP1 depletion (Fig. 4a, b). As expected, ASPP2 depletion induces ZEB1 expression in HCT116 cells (Fig. 4b).

**Fig. 5:**
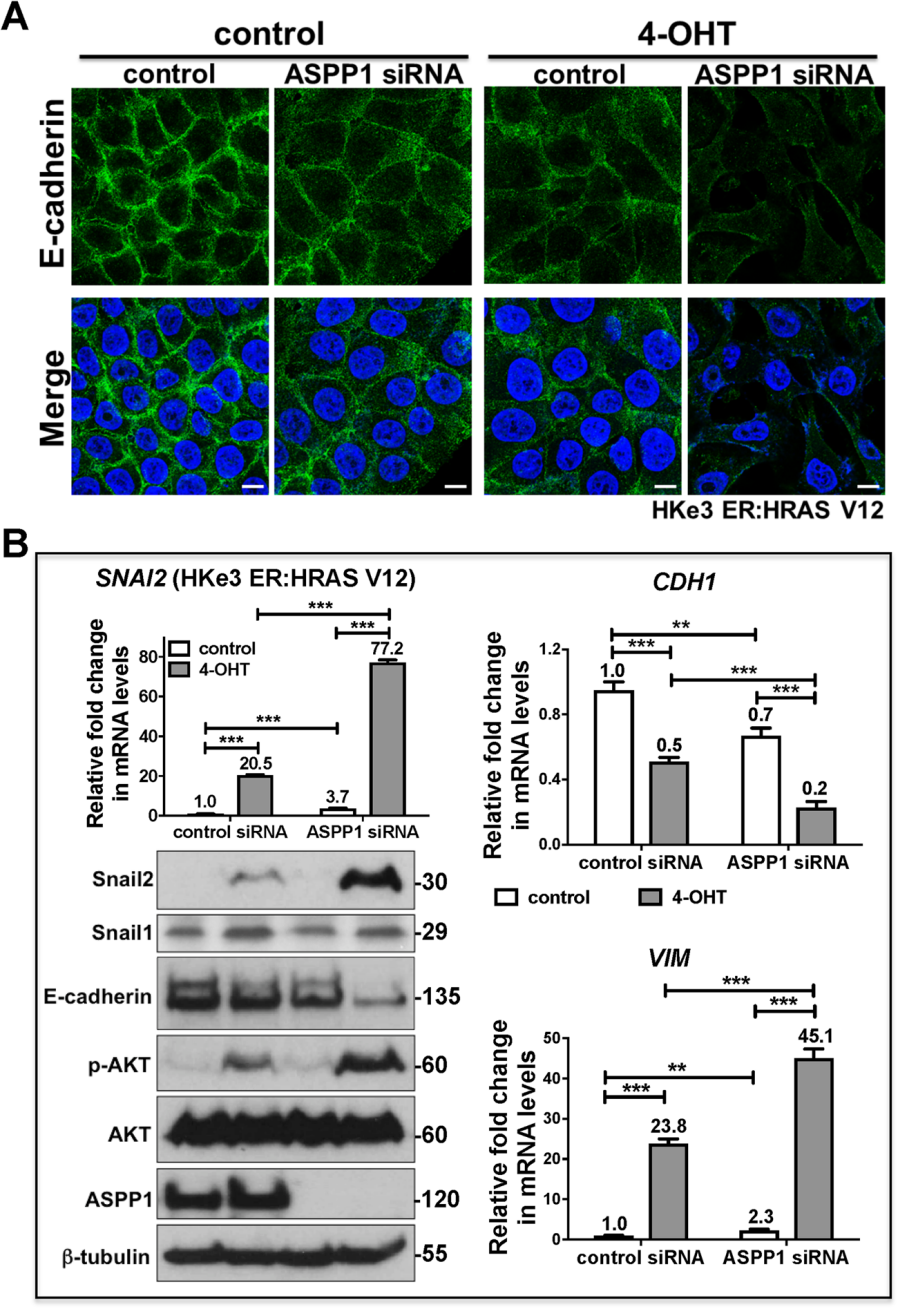
ASPP1 deficiency promotes oncogenic RAS-induced EMT. **a** Immunofluorescence staining of E-cadherin (green) in HKe3 ER:HRAS V12 cells with indicated treatments. TO-PRO-3 (blue) was used to stain nuclei. Scale bars: 10 μm. **b** Fold change in mRNA levels of *SNAI2* (Snail2), *CDH1* (E-cadherin) and *VIM* (Vimentin) in HKe3 ER:HRAS V12 cells with indicated treatments. **P < 0.01. ***P < 0.001. Scores above the bars are relative levels when compared with control cells. Protein expression of Snail1, Snail2, E-cadherin, phospho-AKT (p-AKT), AKT and ASPP1 in HKe3 ER:HRAS V12 cells with indicated treatment. β-tubulin was used as a loading control.

Next, we further validated our results in HKe3 ER:HRAS V12, in which HRASV12 expression is induced by 4-OHT^26,27^. HKe3 is an isogenic derivative of HCT116 with the wild-type HRAS allele generated by genetic disruption of HRAS G13D^28^. Knockdown of ASPP1 alone resulted in a reduction in E-cadherin protein and mRNA levels; however, when ASPP1 is depleted together with 4-OHT treatment this leads to a synergistic effect and E-cadherin (*CDH1*) levels are significantly reduced (Fig. 5a, b). Further, mRNA levels of *SNAI2* (Snail2) are increased upon both RAS-activation and ASPP1 knockdown, however together the treatment increases *SNAI2* levels over 3-fold. *VIM* (Vimentin) levels are 10-fold higher upon RAS-activation compared to ASPP1 knockdown, but both are significantly increased; when both are used together levels of *VIM* double compared to RAS-activated (Fig. 5b). Taken together, this data suggest downregulation of ASPP1 induces EMT in CRC cells and this induction could be enhanced in the presence of active RAS.

### ASPP1 inhibition induces expression of Snail2 via NF-κB pathway

**Fig. 6:**
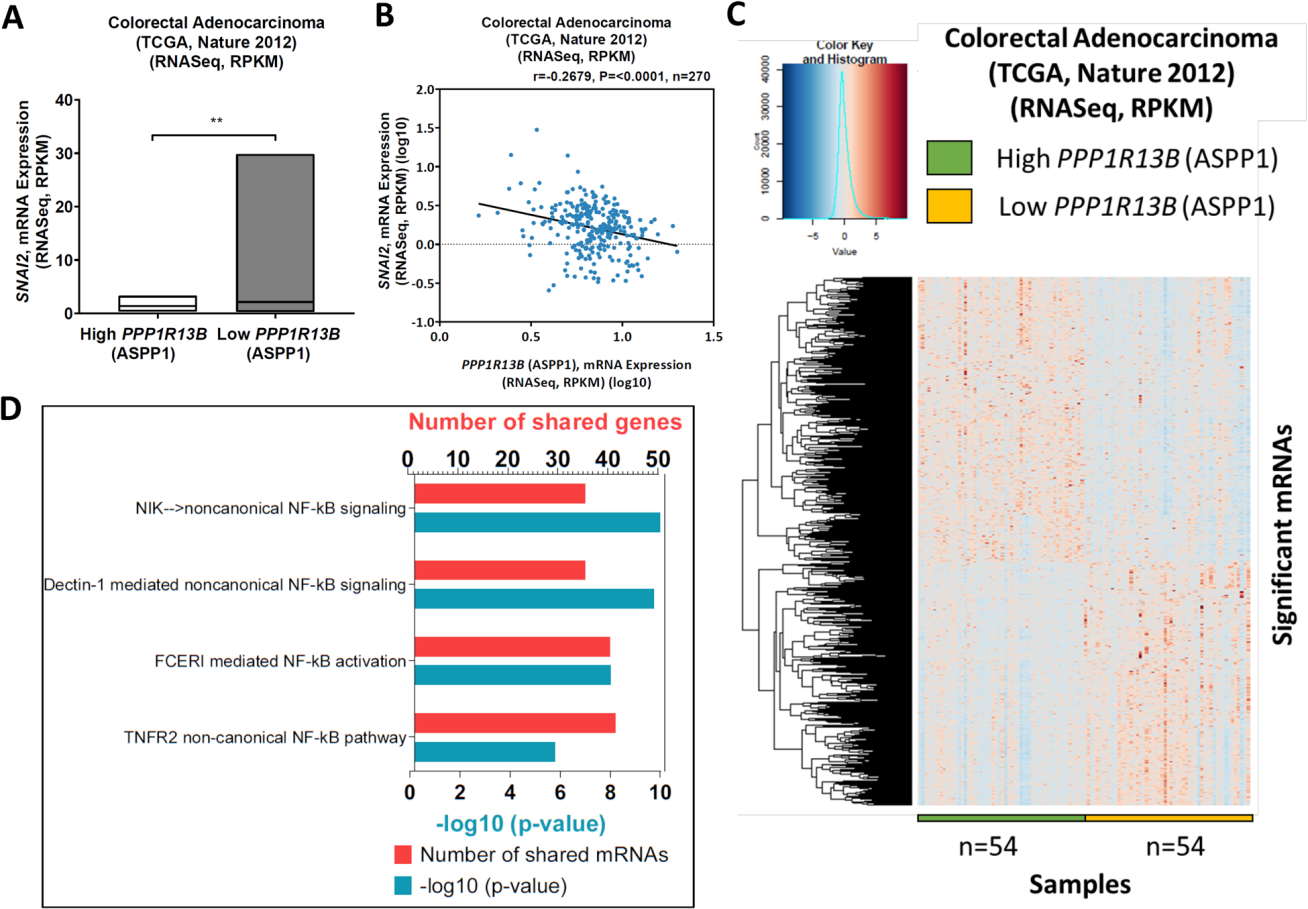
TCGA analysis reveals a link between ASPP1 status and Snail2 expression as well as NF-κB signalling in CRC samples. **a** Bar plot shows the *SNAI2*, mRNA expression between high and low ASPP1 groups in colorectal adenocarcinoma (TCGA, Nature 2012) data. **P < 0.01. **b** The scatter plot for the correlation between *SNAI2*, mRNA expression (RPKM) (log10) and *ASPP1*, mRNA expression (RPKM) (log10) (Pearson’s correlation (r) = −0.2679 and *P* = <0.0001). **c** Heat-maps of the columns indicate each individual sample in high and low PPP1R13B (ASPP1) groups across each mRNA in TCGA colorectal adenocarcinoma data, which obtained from cBioportal website. Rows indicates mRNA expressions (RNASeq, RPKM). Dark blue colour indicates low expression of mRNA and dark pink colour illustrates highly expressed mRNAs. Unpaired *t*-test was analysed to find the significantly different mRNAs in colorectal adenocarcinoma (TCGA, Nature 2012) dataset. *n* represents the number of samples in each group. **d** The genes that were upregulated in high ASPP1 group was analysed in ToppGene Suite website to show which pathways are regulated. Histogram shows NF-κB related pathways. *Y*-axis shows the pathways and top x-axis and bottom x-axis show the number of shared mRNAs in each pathway and −log10 (*P* value), respectively.

The correlation between ASPP1 (*PPP1R13B*) and Snail2 (*SNAI2*) was further confirmed by analyzing TCGA data. Low ASPP1 (*PP1R13B*) expression was significantly correlated with high levels of Snail2 (*SNAI2*) (Fig. 6a, b) in the colorectal adenocarcinoma RNASeq data (TCGA dataset, Nature 2012). In order to find how ASPP1 regulates Snail2 expression, we analysed data from TCGA project. Gene, RNASeq (IlluminaHiSeq) TCGA colorectal adenocarcinoma dataset were obtained from UCSC Cancer Genomics Browser (https://genome-cancer.ucsc.edu/). Significantly differential expressed genes between high and low ASPP1 (*PPP1R13B*) samples are shown in the heatmap in Fig. 6c. To demonstrate whether the significantly positive genes with ASPP1 (*PPP1R13B*) in TCGA dataset are involved in the same pathway, ToppGene Suite (https://toppgene.cchmc.org/) website was used to perform pathway analysis. We found both canonical and noncanonical NF-κB pathways are significantly enriched (Fig. 6d). These analyses suggested that ASPP1 may regulate Snail2 expression via NF-κB pathway.

**Fig. 7:**
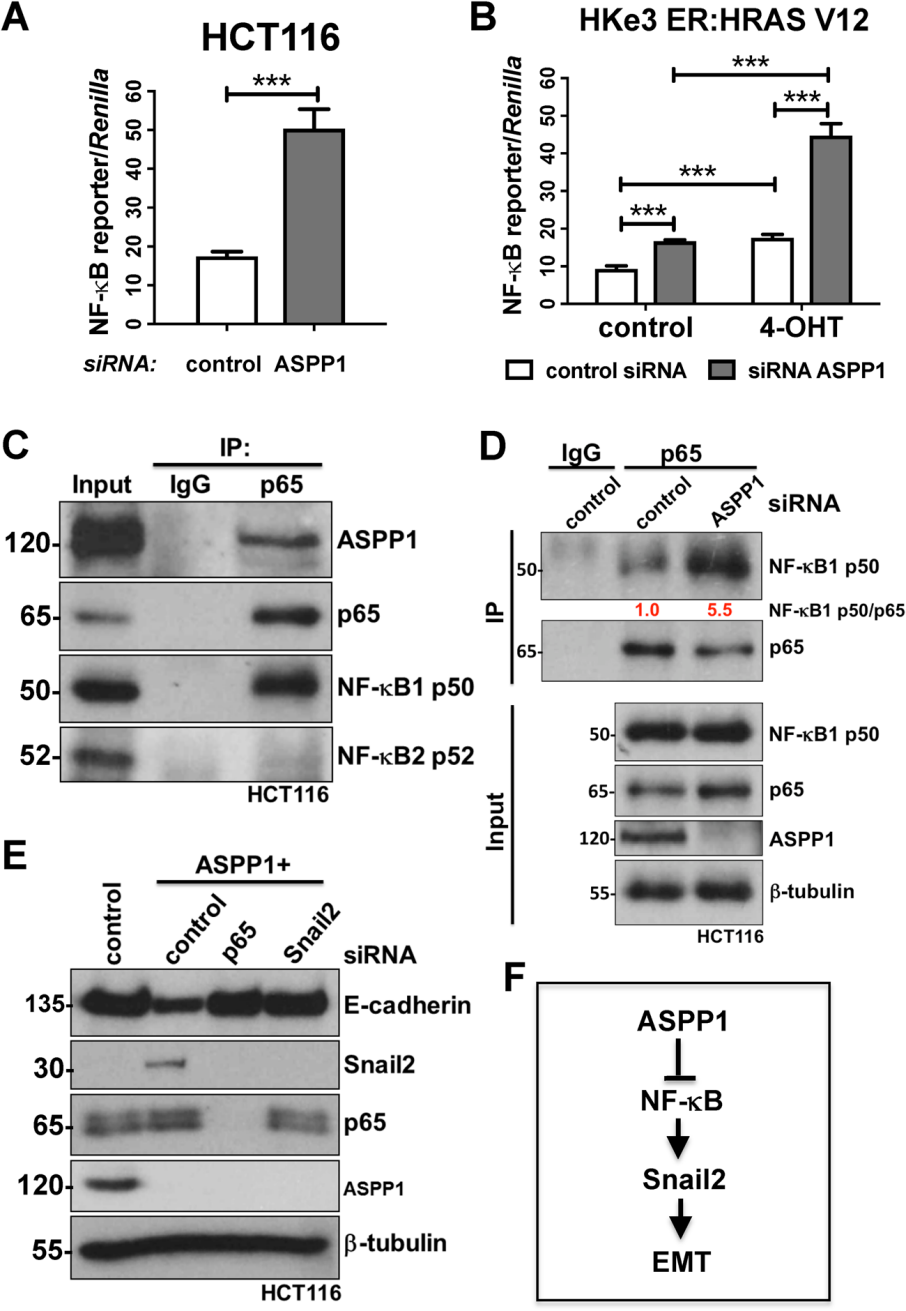
ASPP1 represses Snail2 expression via inhibiting NF-κB pathway. Fold changes in NF-κB reporter activity in HCT116 (**a**) or HKe3 ER:HRAS V12 cells (**b**) with the indicated treatments. NF-κB reporter luciferase readings normalized to *Renilla* in control cells were used to set the baseline value at unity. Data are mean ± s.d. *n* = 3 samples per group. **P < 0.01. ***P < 0.001. **c** Total cell lysates from HCT116 cells were immunoprecipitated (IP) with an anti-p65 antibody or control IgG. ASPP1, p65, NF-κB1 p50 and NF-κB2 p52 levels are indicated. **d** HCT116 cells transfected with control or ASPP1 RNAi were immunoprecipitated with an anti-p65 antibody or control IgG. NF-κB1 p50, p65 and ASPP1 levels are indicated. β-tubulin was used as a loading control. Scores under the bands are relative levels when compared with control cells (1.0). **e** Protein expression of E-cadherin, Snail2, p65 and ASPP1 in HCT116 cells with indicated treatment. β-tubulin was used as a loading control. **f** Diagram showing ASPP1 represses EMT via inhibiting NF-κB-Snail2 pathway in CRC.

Indeed, ASPP1 has been previously demonstrated to bind with NF-κB^29–32^, which is one of the key pathways that can directly bind the promoters of EMT-TFs and induce their expression^33^. To further confirm the role of the NF-κB in these processes we utilized NF-κB reporter assays in HCT116 and HKe3 ER:HRAS V12 cells. Significantly, NF-κB activity in HCT116 transfected with ASPP1 siRNA increased nearly 3-fold compared to control siRNA group (Fig. 7a). In HKe3 ER:HRAS V12 cells, both ASPP1 knockdown and RAS-activation were sufficient to significantly increase NF-κB activity, however RAS-activation together with ASPP1 knockdown over doubled the effect of either treatment-alone (Fig. 7b). To investigate this further, we used an immunoprecipitation assay to try and determine whether ASPP1 could bind with NF-κB p65. In HCT116 cells, NF-κB p65 was immunoprecipitated with ASPP1 and NF-κB1 p50, but not NF-κB2 p52 (Fig. 7c). To further elucidate the role of these proteins, we knocked down ASPP1 in HCT116 cells and conducted a co-immunoprecipitation, we saw a significant increase in the binding between NF-κB1 p50 and p65 upon ASPP1 depletion (Fig. 7d). Finally, to confirm whether ASPP1 regulates Snail2 via NF-κB pathway, in HCT116 cells transfected with ASPP1 RNAi, we depleted Snail2 or p65 simultaneously. Knockdown of ASPP1 induced EMT in HCT116 cells, visible as decreased E-cadherin and increased Snail2 protein levels. The depletion of NF-κB p65 completely abolished the increase in Snail2, and restored E-cadherin expression (Fig. 7e). Depletion of Snail2 (*SNAI2*) was sufficient to restore the epithelial phenotype of HCT116 cells, demonstrated by the western blot of E-cadherin (Fig. 7e). Taken together, these findings suggest that ASPP1 knockdown induces EMT via NF-κB pathway mediated regulation of Snail2 (Fig. 7f).

## Discussion

Colorectal cancer (CRC) has the third highest incidence of malignant tumors and was globally the second leading cause of cancer death in 2018^34^. Development of CRC is a result of interactions between genetics and environmental factors^35^. The RAS family (*KRAS*, *HRAS*, *NRAS*), are frequently mutated in human cancer, approximately 25% of human malignances harbor *RAS* mutations^35^. In CRC, *KRAS* and *NRAS* mutations are found in approximately 44.7% and 7.5% of cases respectively^35^ and RAS has not only been found to drive tumour progression but also is key in tumour maintenance^36–38^. Lifestyle factors, including smoking, diet, lack of exercise, combined with an aging population all contribute to an increased risk of CRC^39^. A number of advances have been made in recent years to both treatment and diagnosis; however, despite new treatments doubling survival for advanced disease^39^, prognosis for metastatic disease is still poor^40^. CRC frequently metastasizes to both the lungs and the liver, and this is a leading cause of treatment failure with up to 50% of CRC patients developing metastatic liver disease after resection of the primary tumour^41^.

The role of ASPP1 in cancer has been reported in several studies^21,22,25,42–44^. However, the relationship between ASPP1 and metastasis has never been reported in CRC. In our study, we found that nuclear ASPP1 is expressed at low levels in CRC patients and correlates with CRC patients TMN clinical stages (stages III + IV vs. I + II) and lymph node metastasis. CRC is typically staged based on a system established by the American Joint Committee on Cancer called the TNM staging system. The tumor is found in nearby lymph nodes from Stage III and distant organs from Stage IV. In the commercial available colorectal tissue microarray used in this study, there are only 3 cases of Stage IV CRC samples, which won’t allow us to directly check is the relationship between ASPP1 expression and distant metastasis. However, in mouse models, we demonstrated that loss of ASPP1 increased metastatic potential of CRC cells to the lungs. These corroborated the in vitro findings, which demonstrated, using Transwell migration and invasion assays that loss of ASPP1 increased invasion and migration in CRC.

The mechanisms underlying metastasis are complicated, from epithelial cell invading locally through surrounding extracellular matrix (ECM) and stromal cell layers, to resuming their proliferative programs at metastatic sites. These complex biological processes are regulated by molecular pathways in cancer cells and cell non-autonomous interactions between carcinoma cells and non-neoplastic stromal cells^14^. EMT is a dynamic process, where epithelial cells convert to a mesenchymal phenotype and this process has been widely implicated in cancer progression^13,14^. EMT can be induced in a number of ways, including by pleiotropically acting EMT-TFs including *SNAI1*/*2* (Snail1/2), *ZEB1*/*2* and *TWIST1*/*2*^15^, these have been shown to have a role in cancer. Snail2 (*SNAI2*), in particular, has been reported to have a key role in the invasion and migration of cancer^16,45,46^ and we have previously demonstrated it can promote EMT in RAS-active colorectal cancer cells^27^.

The NF-κB pathway consists of five transcription factors and these regulate a number of cellular processes, such as proliferation and apoptosis^47^. Constitutive NF-κB activity has been reported in human cancers^29,47,48^, as a result of inflammatory microenvironment and oncogenic mutations^47^, and has been linked to cancer progression and promoting an invasive phenotype^49–52^. Further it has been shown to mediate EMT-TFs, ZEB1 and TWIST1, to regulate EMT^53^. Although ASPP1 has been shown to bind with NF-κB^30–32,54^, these mechanisms haven’t been previously described in cancer. Here we show using analysis of CRC TCGA datasets that reduced ASPP1 expression correlates with increased *SNAI2* expression and pathway analysis showed that low ASPP1 expression resulted in NF-κB activation. We observed that downregulating ASPP1 induces NF-κB activation, potentially by enhancing the complex formation between NF-κB1 p50 and NF-κB p65. Together, these data suggest that ASPP1 inhibition induces expression of Snail2 via NF-κB pathway (Fig. 7f).

Taken together, we demonstrated that ASPP1 expression was reduced in CRC and low ASPP1 expression was correlates with higher clinical stage. Our study also identifies a new mechanism showing that downregulated ASPP1, could increase Snail2 to promote invasion and migration in colorectal cancer via active NF-κB. Further, downregulated ASPP1 could cooperate with active RAS could facilitate EMT, invasion and migration via upregulating expression of Snail2. Our research provides a valuable indication that ASPP1 could be a prognostic indicator of CRC and patients with low ASPP1 levels could be more suitable for therapy with NF-κB inhibitors^55,56^. These results may offer new information for the development of novel drugs in the treatment of CRC.

## Methods

### Colorectal cancer tissue microarray and immunohistochemistry (IHC)

A colorectal tissue microarray (HColA180Su11; Shanghai Outdo Biotech, Shanghai, China) with 86 matched pairs of primary CRC samples and adjacent normal tissues were purchased for IHC analysis. All procedures were approved by the Ethical Committee of Tongji Hospital. Informed consent was obtained from all subjects. The tissue section was de-waxed, rehydrated and incubated with 3% hydrogen peroxide to block endogenous peroxidase activity for 30 min. After microwave antigen retrieval, sections were blocked and then incubated overnight at 4 °C with a primary antibody against ASPP1 (1:100, HPA006394, Sigma). Sections were then incubated with secondary antibodies for 60 min at room temperature. After washing with TBS three times, the slides were stained with 3,3-diaminobenzidine and counterstained with hematoxylin. Counterstained by haematoxylin, the slides were dehydrated in graded alcohol and mounted. For each section, five random non-overlapping fields containing at least 200 cells per field were observed and scored based on the percentage of positively stained cells (0–100%) and the staining intensity (score 0 for negative, 1 for weak staining, 2 for moderate staining, and 3 for strong staining). The immunoreactive score (IRS) was calculated with the formula, the percentage of positive cells × the staining intensity × 100, to produce a value between 0 and 300. Patients were divided into low and high expression groups according to the median IRSs.

### Cell culture, reagents and transfections

HKe3 ER:HRAS V12, HCT116 and firefly luciferase tagged HCT116 cells were cultured in DMEM (Fisher Scientific UK, 11594446) supplemented with 10% FBS (Invitrogen, 10270106). MCF10A ER: HRAS V12 cells were maintained in a 1:1 mixture of DMEM and Ham’s F12 medium (Fisher Scientific UK, 11524436) supplemented with 5% horse serum (Gibco, 26050088), 20 ng/ml EGF (Bio-techne, 236-EG), 100 ng/ml cholera toxin (Sigma, C8052), 10 μg/ml insulin (Sigma, I1882), 500 ng/ml hydrocortisone (Sigma, H0888) and antibiotics. Cells were kept in a humidified 37 °C incubator with 5% CO_2_ and 95% air. For 3D acini cultures, MCF10A ER:HRAS V12 cells were cultured as previously described^57^ on growth factor-reduced Matrigel (BD Biosciences, 354230). 4-Hydroxytamoxifen (4-OHT) was purchased from Sigma-Aldrich (H6278). No mycoplasma contamination was detected in the cell lines used.

MCF10A ER:HRAS V12 cells were transfected with the indicated siRNA oligos at a final concentration of 37.5 nM using Lullaby (OZ Biosciences, LL71000), according to the manufacturer’s instructions. All other cell lines were transfected with the indicated siRNA oligos at a final concentration of 35 nM using Dharmafect 1 reagent (Dharmacon, T-2001–03). Short interfering RNA (siRNA) oligos against ASPP1 (MQ-010492-01-0002) were purchased from Dharmacon (Lafayette, CO, USA). Sequences are available from Dharmacon or upon request. As a negative control, we used siGENOME RISC Free siRNA (Dharmacon).

Stable knockdown of human ASPP1 was carried out using pLVX-shRNA-mCherry-Hygro lentiviral expression plasmid. The ASPP1 short hairpin RNA (shRNA) plasmid was designed to target the three independent shRNA constructs. Target sequences are:

shRNA-1, 5‘-GCAACGAACTCAGAGAAATGT-3’;

shRNA-2, 5‘-GGTTGGGAATCCACGTGTTGA-3’;

shRNA-3, 5‘-GCAATCTGTCTGCTGAAATAG-3’.

### Western blot analysis

Western blot analysis was performed with lysates from cells or tissues with urea buffer (8 m urea, 1 m thiourea, 0.5% CHAPS, 50 mm DTT and 24 mm spermine). For immunoprecipitations, the cells were lysed for 30 min at 4 °C in pNAS buffer [50 mm Tris/HCl (pH 7.5), 120 mm NaCl, 1 mm EDTA and 0.1% Nonidet P-40], with protease inhibitors. Indicated antibodies and immunoglobulin G (IgG) agarose were added to the lysate for 16 h at 4 °C. Immunoprecipitates were washed four times with cold PBS followed by the addition of SDS sample buffer. The bound proteins were separated on SDS polyacrylamide gels and subjected to immunoblotting with the indicated antibodies. Primary antibodies were from: Sigma (ASPP1, HPA006394; ASPP2, HPA021603), Santa Cruz Biotechnology (ZEB1, sc-25388; E-cadherin, sc-21791; Snail2, sc-166476), Abcam (β-tubulin, ab6046; NF-κB1 p50, ab32360; NF-κB2 p52, ab174482; ZEB2, ab138222), Cell Signaling Technology (phospho-AKT, 9271; AKT, 4685; Snail1, 3879; Snail2, 9585; NF-κB p65, 8242), BD Transduction Laboratories (E-cadherin, 610405). Signals were detected using an ECL detection system (GE Healthcare) (Chicago, IL, USA) or an Odyssey imaging system (LI-COR), and evaluated by ImageJ 1.42q software (National Institutes of Health) (Berhesda, MD, USA).

### qRT-PCR

The real-time RT-PCR was carried out using gene-specific primers (QuantiTect Primer Assays, Qiagen) for *SNAI1*, *SNAI2*, *ZEB1*, *ZEB2*, *TWIST1*, *CDH1*, *VIM*, *GAPDH* or *ACTN* with QuantiNova SYBR Green RT-PCR kits (Qiagen). Relative transcript levels of target genes were normalised to *GAPDH* or *ACTN* mRNA level.

### Transwell migration and Matrigel invasion assays

For the Transwell migration assay, Transwell membranes (8-μm pore size, 6.5-mm diameter; Corning Costar, 3422) were used. The bottom chambers of the Transwell were filled with migration- inducing medium (with 30% FBS). The top chambers were seeded with 1.5 × 10^5^ live serum-starved control or ASPP1 shRNA HCT116 cells per well. After 48 h, the filters were fixed with 4% paraformaldehyde for 10 min at room temperature; subsequently, the cells on the upper side of the membrane were scraped with a cotton swab. Similar inserts coated with Matrigel (Corning, 354480) were used to determine invasive potential in invasion assays. Filters were stained with crystal violet for light microscopy. Images were taken using an Olympus inverted microscope and migratory cells were evaluated by ImageJ 1.42q software (National Institutes of Health, United States).

### Immunofluorescence microscopy

Cells were fixed in 4% PBS (Fisher Scientific UK, 12579099)-paraformaldehyde for 15 min, incubated in 0.1% Triton-X-100 (Fisher Scientific UK, 11471632) for 5 min on ice, then in 0.2% fish skin gelatin (Sigma, G7041) in PBS for 1 h and stained for 1 h with an anti-E-cadherin (1:100, Santa Cruz sc-21791, mouse monoclonal 67A4) antibody. Protein expression was detected using Alexa Fluor 488 (1:400: Fisher Scientific UK) for 20 min. TO- PRO-3 (Invitrogen, T3605: 1:1000) was used to stain nucleic acids. For immunofluorescence staining of 3D cultures from MCF10A ER:HRAS V12 cells, acini were fixed with 4% paraformaldehyde for 40 min, permeabilized in 0.5% Triton X-100 for 10 min on ice and stained with Rhodamine-phalloidin (Molecular Probes, R415) for 1 h at room temperature. Acini were counterstained with DAPI. Samples were observed using a confocal microscope system (Carl Zeiss LSM 510 or LSM 710, or Leica SP8). Acquired images were analyzed using Photoshop (Adobe Systems, United States) according to the guidelines of the journal.

### TCGA data mining and pathway analysis

Colorectal adenocarcinoma (TCGA, Nature 2012), RNASeq, RPKM, mRNA expression was obtained from the cBioPortal for Cancer Genomics website (http://www.cbioportal.org/). The colorectal adenocarcinoma samples were separated into high and low *PPP1R13B* (ASPP1), mRNA expression, by selecting the top and bottom 20% of samples for high and low ASPP1 expression, respectively. Then an unpaired *t*-test was performed to find the significantly different (*P* < 0.05) mRNAs between high and low ASPP1 sample groups (all done using *R*, version 3.4.4).

To explore the pathways represented in the colorectal adenocarcinoma TCGA mRNA data analysis, ToppGene Suite (https://toppgene.cchmc.org/) website was used. In TCGA mRNA data, 4319 mRNAs were significantly upregulated in high ASPP1 sample group compared to the low ASPP1 group. The identified 4319 mRNAs in TCGA gene data were analysed with ToppGene Suite website to detect functional enrichment of the genes on pathway analysis. The identified NF-κB related pathways in pathway analysis with the number of shared mRNAs and *P*-value were plotted with histogram plot in GraphPad Prism V8.2.1.

GraphPad Prism V8.2.1 was used to plot bar plot to identify mRNA expression of *SNAI2* between high and low ASPP1 groups in colorectal adenocarcinoma (TCGA, Nature 2012) dataset. Moreover, the correlation between *SNAI2* and ASPP1 mRNA expression within all colorectal adenocarcinoma samples was analysed by Pearson’s correlation analysis in GraphPad Prism V8.2.1.

### Luciferase reporter assay

HCT116 and HKe3 ER:HRAS V12 cells were transfected with the indicated siRNAs for 48 h in 24-well plates, followed by 24 h transfection with 250 ng of NF-κB reporter and 10 ng of phRL-CMV (Promega, E2261), which constitutively expresses the *Renilla* luciferase reporter. One day before the measurement of luciferase activity 100 nM 4-OHT was added. Finally, the transcription assay was carried out using the Dual-luciferase® reporter assay system (Promega, E1960) following the manufacturer’s protocol.

### Tumorigenicity and metastasis experiment

For lung metastasis, 1 × 10^6^ cells were injected into the tail vein of 8 weeks old male nude mice anesthetized with 2% isoflurane gas. Two weeks later, Images of Lung fluorescence signals were acquired by IVIS Lumina LT series III (Caliper, MA) with the excitation (640 nm) and emission wavelength (710 nm 845 nm). The mice were injected with D-luciferin, anesthetized with isofluorane, and imaged 5 min after luciferin injection. Fluorescence intensity within specific regions of individual animals was quantified using the region of the interest (ROI) tools in the Live Image 4.5 software (PerkinElmer). Four weeks after injection, mice were euthanized by CO_2_ followed by cervical dislocation. Lungs were removed and fixed in 10% buffer formalin and paraffin embedded. Sections (5 μm) of lungs were stained with hematoxylin and eosin to visualize the pulmonary metastases under a microscope. All animal experiments were performed in accordance with a protocol approved by the Ethics Committee of Tongji Hospital, Tongji Medical College, Huazhong University of Science and Technology, China.

### Blinding, randomization, statistical analysis and repeatability of experiments

IHC scoring was conducted with the researcher blind to the treatment. The animal studies were randomized and blinded according to the protocol approved by Ethics Committee of Tongji Hospital, Tongji Medical College, Huazhong University of Science and Technology, China. The sample size was estimated in light of a retrospective analysis of our previous studies^26^. Each experiment was repeated at least twice. Unless otherwise noted, data are presented as mean ± s.d., and a two-tailed, unpaired Student’s *t*-test was used to compare two groups for independent samples. Chi-square test or Fisher exact test were used to evaluate the relationship of ASPP1 expression and clinical parameters of CRC. Correlation between the expression of ASPP1 and Snail2 was analysed using Pearson’s correlation. Statistical analysis was conducted using SPSS version 19.0 (Endicott, NY, USA). Unpaired *t*-test was performed for TCGA data analysis in *R* version 3.4.4 or in GraphPad prism V8.2.1. *P* < 0.05 was considered statistically significant.

## Supplementary information

Supplementary Text

Supplementary Figure S1

Supplementary Figure S2

Supplementary Figure S3
