# Supplementary material for: ASPP1 deficiency promotes epithelial-mesenchymal transition, invasion and metastasis in colorectal cancer
Source: Cell Death Dis. 2020 Apr 8;11(4):224. doi: 10.1038/s41419-020-2415-2 (PMC7142079; doi:10.1038/s41419-020-2415-2)
Supplement: Supplementary file 1 — Supplementary Text [file 41419_2020_2415_MOESM1_ESM.docx]

**Supplementary Figure Legends**

**Supplementary Fig. 1** Expression of ASPP1 is decreased in colorectal cancer (CRC). **a** Representative ASPP1 expression in paired human CRC and adjacent normal tissues. Scale bars: 500 μm. Images in the upper panel are also shown in Fig. 1a. **b** Comparison of ASPP1 expression in 86 paired CRC and adjacent normal tissues in nucleus (*P* = 0.0007) or cytoplasm (*P* < 0.0001), respectively.

**Supplementary Fig. 2** Downregulation of ASPP1 with shRNAs. Protein expression of ASPP1 in HCT116 cells with indicated treatment. β-actin was used as a loading control.

**Supplementary Fig. 3** Downregulation of ASPP1 promotes cell invasion *in vitro.* **a** Representative 3D confocal images of MCF10A ER:HRAS V12 cells cultured in Matrigel with indicated treatment. MCF10A ER:HRAS V12 cells were transfected with control or ASPP1 siRNA for 5 days, followed by 100 nM 4-OHT treatment for 1 day. Spheres were stained for F-actin with Rhodamine-phalloidin (yellow) and DAPI (blue). Scale bars: 40μm. **b** Protein expression of ASPP1, ASPP2 and phospho-ERK (p-ERK) in MCF10A ER:HRAS V12 cells with indicated treatment. β-tubulin was used as a loading control.
