# Supplementary figures and images for: ASPP1 deficiency promotes epithelial-mesenchymal transition, invasion and metastasis in colorectal cancer
Source: Cell Death Dis. 2020 Apr 8;11(4):224. doi: 10.1038/s41419-020-2415-2 (PMC7142079; doi:10.1038/s41419-020-2415-2)

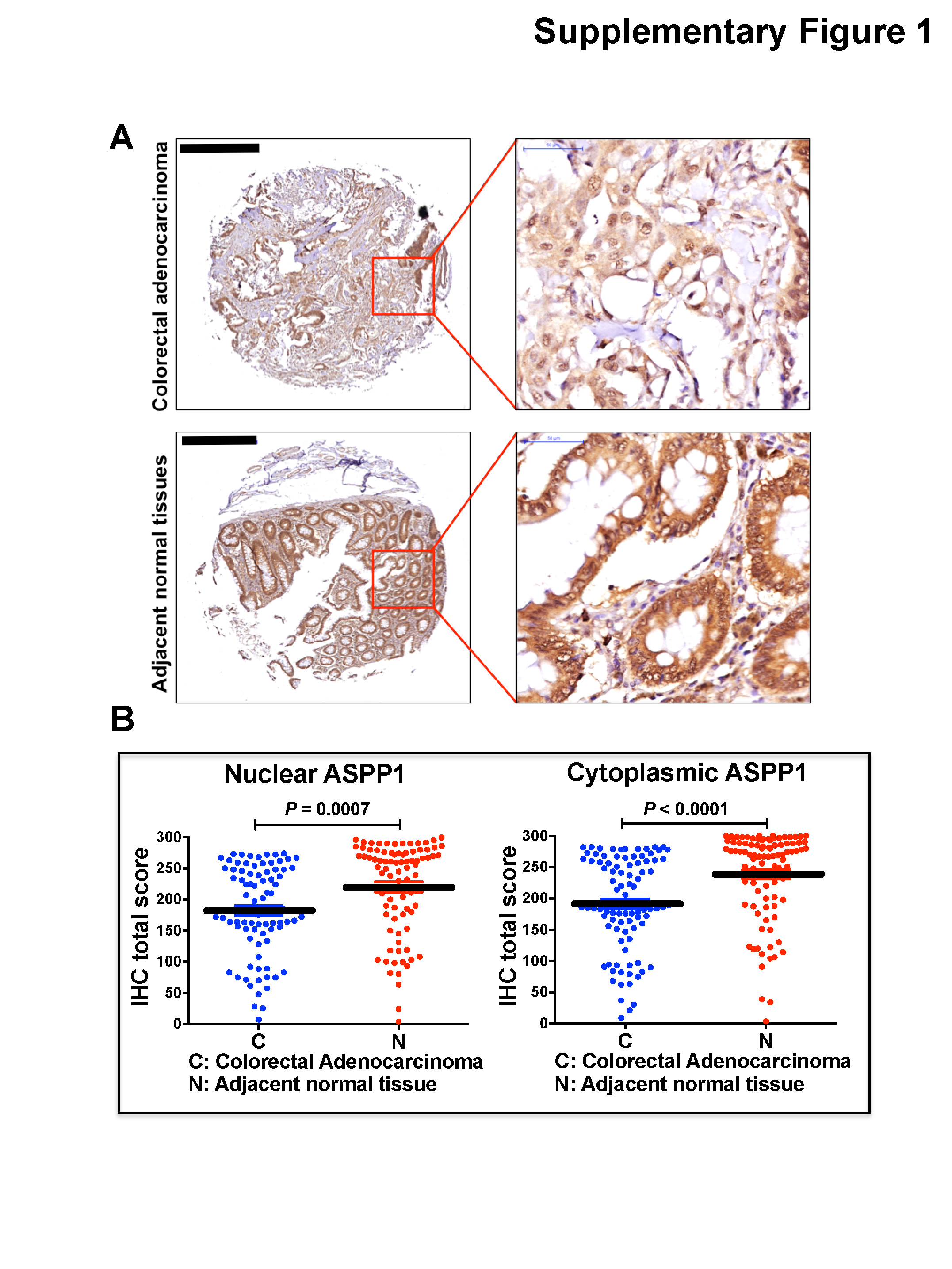

Supplement: Supplementary file 2 — Supplementary Figure S1 [file 41419_2020_2415_MOESM2_ESM.tif]

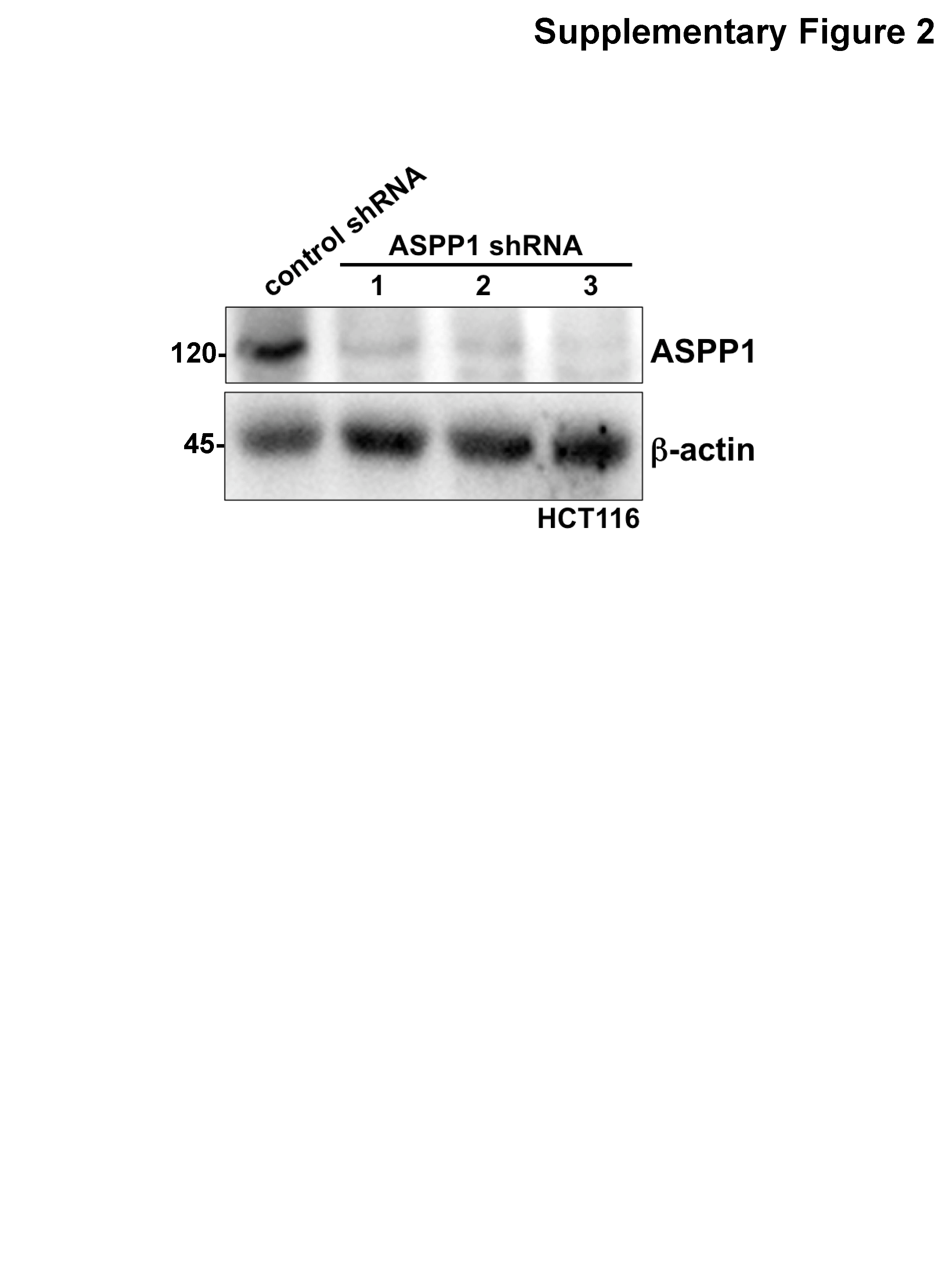

Supplement: Supplementary file 3 — Supplementary Figure S2 [file 41419_2020_2415_MOESM3_ESM.tif]

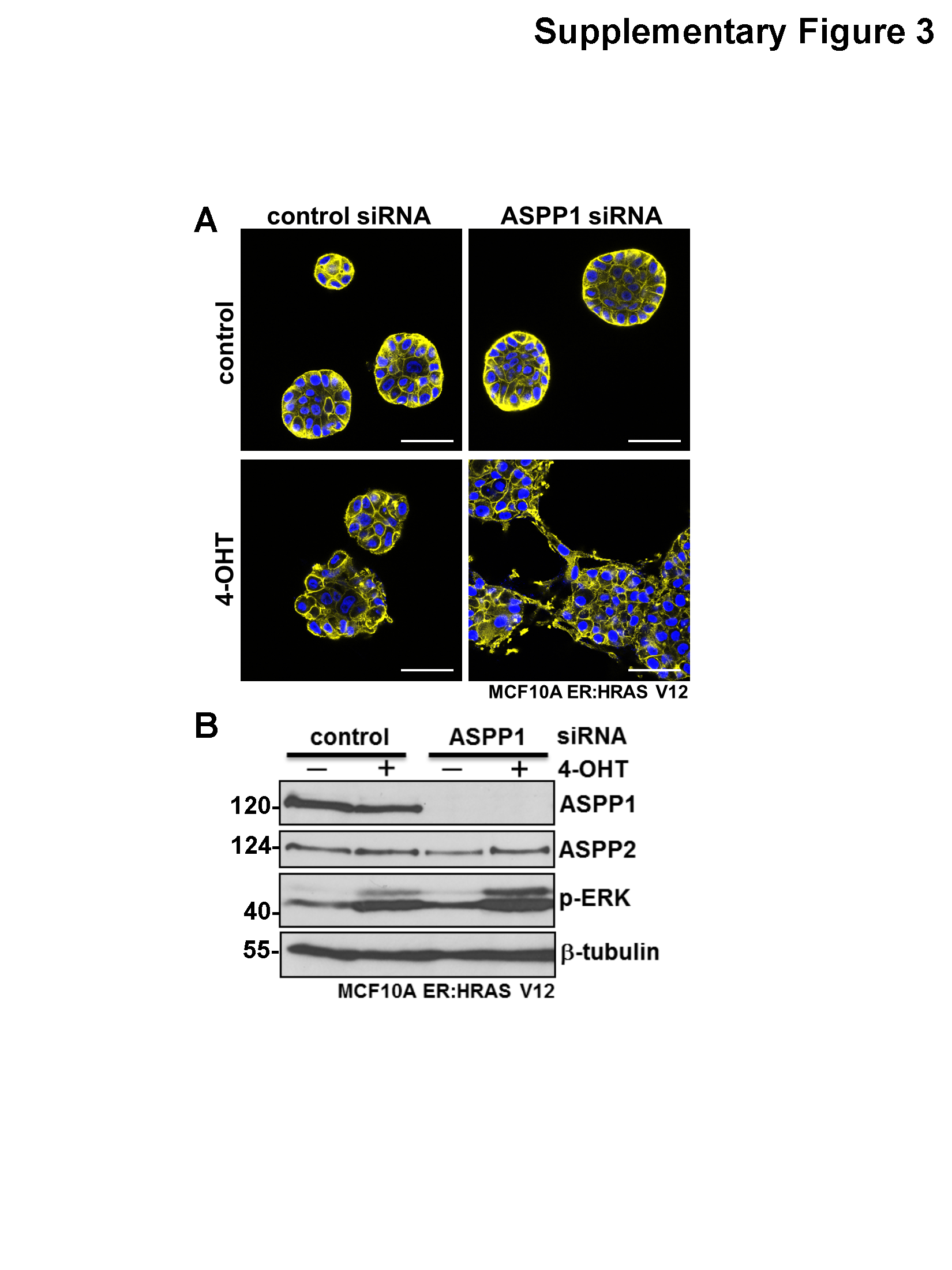

Supplement: Supplementary file 4 — Supplementary Figure S3 [file 41419_2020_2415_MOESM4_ESM.tif]
